# Electrical Activity of the Diaphragm (Edi) Metrics in Premature Infants Receiving Invasive Mechanical Ventilation Versus Noninvasive Respiratory Support

**DOI:** 10.7759/cureus.102317

**Published:** 2026-01-26

**Authors:** Sarah Fleishaker, Tatiana Nuzum, Rasik Shah, Sean Bailey, Pradeep Mally

**Affiliations:** 1 Pediatrics, NYU Langone Health, New York City, USA; 2 Pediatric Pulmonology, NYU Langone Health, New York City, USA

**Keywords:** bronchopulmonary dysplasia, edi metrics, electrical activity of the diaphragm, neonatology, pediatrics, pulmonology

## Abstract

Background: In neonatology, many of our patients require respiratory support for a variety of pathologies. However, there is little data regarding objective parameters to help guide this support. Decisions regarding the optimal level of support for each patient remain largely subjective. The electrical activity of the diaphragm (Edi) is a measure of neural respiratory drive and inspiratory load. This could serve as a useful tool to provide objective data regarding respiratory status, which in turn may aid in decision-making about optimal respiratory support.

Objectives: The primary objective of this study was to compare Edi metrics in infants who required invasive mechanical ventilation with those who remained on noninvasive modes of ventilation. The secondary objective was to compare the rates and severity of bronchopulmonary dysplasia (BPD) in the two groups.

Design/methods: This was a prospective observational pilot study. Infants admitted to the neonatal ICUs (NICUs) at Hassenfeld Children’s Hospital and Bellevue Hospital Center, born at a gestational age of 26-32 weeks and weighing >500 grams, were eligible for the study. An Edi catheter (Maquet Critical Care AB, Solna, Sweden) was placed within the first 24 hours of life and remained in place for 72 hours. Subjects were weaned or escalated on their respiratory support based on current unit practice guidelines. They were separated into two groups: those who required invasive mechanical ventilation and those who did not. Paired Student's t-tests, chi-square tests, and Mann-Whitney tests were used to evaluate statistical significance (p ≤ 0.05).

Results: A total of 29 subjects were enrolled: 15 in the intubated group and 14 in the noninvasive group. Edi minimum was significantly lower, and delta Edi (the difference between Edi peak and minimum) was higher in the non-intubated group. There were no statistical differences in Edi peaks between the groups, although there was a trend toward higher peaks in the noninvasive group. Additionally, BPD outcomes were not statistically different.

Conclusions: The lower Edi minimum in the non-intubated group suggests less severe lung disease and milder respiratory distress syndrome (RDS), requiring less stimulus and external support to generate adequate distending pressure to maintain alveolar patency at end-expiration. The higher delta Edi indicates a greater intrinsic ability to generate an adequate tidal volume for ventilation. There is wide variation in practice regarding modes of ventilation, both invasive and noninvasive, with no clear best practice. We lack objective data to guide our practice. This study is the first to examine Edi metrics as a monitoring tool to assess the degree of disease severity and sufficiency of support provided. Our results suggest that using Edi metrics may provide more data to help standardize and guide clinical practice.

## Introduction

In the field of neonatology, many of our patients require respiratory support for a variety of pathologies. We know that invasive mechanical ventilation, particularly prolonged periods of mechanical ventilation, is associated with several adverse outcomes such as ventilator-associated pneumonia, airway trauma, and bronchopulmonary dysplasia (BPD) [1]. BPD remains the most common complication associated with prematurity, and its prevalence increases with decreasing gestational age and birth weight. In the United States (U.S.), approximately 35% of extremely low gestational age newborns develop BPD [2]. Furthermore, BPD is associated with neurodevelopmental impairments [3]. For these reasons, along with improved noninvasive clinical respiratory management, there has been a shift in respiratory management from the routine use of invasive respiratory support to an increased utilization of noninvasive respiratory support for cohorts of premature infants.

It has also been established that over-supporting respiratory mechanics, which use various modes of ventilators, can result in volutrauma, barotrauma, and subsequent inflammation. Conversely, under-supporting a premature infant’s respiratory drive and function can result in complications such as apnea, atelectasis, and subsequent inflammation [4], all of which are known precursors of BPD. Therefore, our goal as clinicians is to optimize ventilation by using the least invasive mode of respiratory support that is adequate for ideal lung development, oxygenation, and ventilation. 

Much of the research to date regarding respiratory management in premature neonates has focused on bedside clinical observations and laboratory results because of their ease of use and convenience. However, these predictors individually have proven to be suboptimal in predicting respiratory function, likely because it is improbable that any single predictor can accurately encompass all possible reasons for requiring more respiratory support. Researchers have demonstrated that readiness for extubation in premature neonates remains challenging, with almost 30% of neonates requiring reintubation within seven days after their first extubation attempt [5].

The reason why premature neonates often have complications secondary to prolonged intubation is that there is a high degree of asynchrony between the infant’s breathing and the ventilator’s "breath." This asynchrony is primarily a result of leaks around the endotracheal tube, high respiratory rates, and high variability in the breathing patterns [6]. Suboptimal patient-ventilator interaction is associated with patient discomfort, poor lung aeration, and increased work of breathing, which leads to a subsequent need for higher ventilator settings [7-9]. 

Neurally adjusted ventilator assist (NAVA) is an innovative mode of ventilation that applies ventilator support in proportion to the patient's measured electrical activity of the diaphragm (Edi), which is the largest muscle used during respiration. Therefore, ventilation is controlled directly by the patient’s own neural control of breathing. It can be used for invasive ventilation or noninvasive ventilation. The neural control originates in the respiratory center, and signals are transmitted through the phrenic nerve to trigger Edi, which in turn recruits diaphragmatic motor units, leading to a proportional respiratory breadth. This electrical activity is measured through a special feeding tube (Edi catheter; Maquet Critical Care AB, Solna, Sweden) that also contains a multiple array of esophageal electrodes [10]. These Edi signals can be used as a monitoring tool to assess diaphragmatic and brainstem function and are a validated measure of respiratory drive. The Edi peak is the highest Edi value reached during a breathing cycle and represents the maximum diaphragmatic contraction during inspiration. The Edi minimum is the lowest Edi value reached during a breathing cycle and represents the diaphragmatic activity at the end of expiration. With worsening respiratory pathology, Edi metrics are expected to increase [11]. When used in NAVA mode, a "normal" Edi minimum is <3 µV, while a "normal" Edi peak is anywhere between 5-15 µV. 

While NAVA is approved and has been shown to be an effective and more synchronous mode of ventilation, it is not yet widely used across neonatal ICUs (NICUs). The concern and preoccupation with providing optimal respiratory support for premature infants, specifically determining the need for invasive versus noninvasive ventilation, remains a pressing question in the field. The Edi catheter can be used as a means of monitoring the diaphragmatic signals, even when not in NAVA mode, and can potentially help quantify disease severity and guide clinical management. There are no studies to date examining differences in Edi metrics between infants who require mechanical ventilation and those who do not. When used as a monitoring tool, there are no clear values to indicate the need for increasing respiratory support or the ability to wean respiratory support. It is thus crucial to follow Edi trends in individual cases, which, along with existing clinical and lab parameters, may better help to escalate or wean related respiratory support.

Objectives

The primary objective of this study was to compare Edi metrics (peak and minimum values) between infants who required invasive mechanical ventilation and those who remained on noninvasive modes of ventilation, in order to better understand neural respiratory drive and its relationship to disease severity. The secondary objective was to compare the rates and severity of BPD between the two groups.

## Materials and methods

Materials

The NAVA/Edi catheter is U.S. Food and Drug Administration (FDA)-cleared and intended for administering nutrition, fluids, and medication via the naso-gastroenteric route, as well as aspiration via the naso-gastroenteric route. It also transfers electrical activity (Edi signals) to compatible Servo ventilator systems (Maquet Critical Care AB, Solna, Sweden) that support NAVA and noninvasive NAVA (NIV-NAVA). The NAVA/Edi catheter is FDA approved for neonatal, pediatric, and adult patients.

Methods

This prospective observational pilot study was conducted from September 2021 to January 2022 at two regional perinatal centers in New York City: Hassenfeld Children’s Hospital at NYU Langone Health and NYC Health + Hospitals/Bellevue Hospital Center. Institutional Review Board (IRB) approval was obtained for both sites prior to the study's conduct (i21-01071). Informed consent was obtained for all subjects recruited for the study, and the research was conducted in accordance with ethical guidelines.

All infants admitted to the Hassenfeld Children’s Hospital and Bellevue Hospital Center NICUs, born between 26 and 32 weeks of gestation, with a birth weight greater than 500 grams and requiring invasive or noninvasive respiratory support, were eligible. Exclusion criteria included neonates with congenital anomalies or genetic syndromes that could impact respiratory drive, hypoxic-ischemic encephalopathy requiring therapeutic hypothermia, those requiring high-frequency ventilation, and neonates with a five-minute Apgar score of less than 3 [12].

Relevant patient demographics, clinical events, and vital signs were collected. A 6-Fr Edi catheter was placed within the first 24 hours of life, and monitoring continued for the subsequent 72 hours. Edi placement was confirmed by X-ray per unit policy, as well as by Edi tracing displayed in NAVA mode on the Servo-u® ventilator. As this was an observational study, the clinical team did not see or analyze the Edi metrics; they continued clinical management based on their judgment and following our current unit policies and guidelines. Infants were intubated or remained on noninvasive ventilation (synchronized inspiratory positive airway pressure (SiPAP) or continuous positive airway pressure (CPAP)) based on fraction of inspired oxygen (FiO_2_) requirements, work of breathing, blood gas analysis, and overall clinical status, per unit policy. Respiratory support was subsequently weaned or escalated based on these same parameters. Edi metrics were collected following the 72-hour monitoring period and analyzed in six-hour increments.

Subjects who were intubated during these 72 hours were in the invasive mechanical ventilation group and were compared to those infants who remained on noninvasive respiratory support. The Edi catheter was strictly used as a monitoring tool, and infants were not placed on NAVA mode, as this is not currently our unit’s standard practice.

Paired Student's t-tests and chi-square tests were used to evaluate statistical significance in the outcomes, including Edi metrics and BPD, based on the Jenson criteria.

## Results

A total of 29 subjects were enrolled in this study: 15 required intubation and were in the intubated group, while 14 did not require intubation and were in the noninvasive group. Demographic information of the subjects was collected and evaluated using paired Student’s t-tests and chi-square tests. There were statistically significant differences in birth weights, gestational ages, and one-minute Apgar scores between the two groups (Table 1) [12].

**Table 1:** Patient demographics * mean ± standard deviation, ‡ n (%), † median (interquartile range)

	Intubated group (n=15)	Noninvasive group (n=14)	P-value
Gestational age (weeks) *	28.57 ± 2.2	30.43 ± 1.4	0.03
Body weight (kg) *	1.23 ± 0.35	1.5 ± 0.28	0.04
Female sex ‡	4 (27)	5 (36)	0.60
Betamethasone complete ‡	12 (80)	11 (79)	0.92
1-minute Apgar †	4 (2, 8)	8 (7.25, 8)	<0.01
5-minute Apgar †	9 (7, 9)	9 (8.75, 91)	>0.05

When looking at the Edi metrics using paired Student’s t-tests, the Edi minimum was significantly lower, while the delta Edi (the difference between Edi peak and minimum) was higher in the non-intubated group. There were no statistically significant differences in Edi peaks between the groups, although there was a trend toward higher peaks in the noninvasive group. There were no statistically significant differences in respiratory rates or oxygen requirements (Table 2).

**Table 2:** Edi metrics * mean ± standard deviation Edi: electrical activity of the diaphragm; FiO_2_: fraction of inspired oxygen; min: minimum

	Intubated group (n=15)	Noninvasive group (n=14)	P-value
Edi peak (μV) *	8.8 ± 1.33	9.86 ± 2.55	0.18
Edi min (μV) *	2.08 ± 0.48	1.69 ± 0.32	0.02
Delta Edi (μV) *	6.72 ± 1.09	8.17 ± 2.31	0.04
Respiratory rate (bpm) *	54.74 ± 12.62	48.88 ± 8.99	0.17
FiO_2_ requirement (%) *	32 ± 11	28 ± 4	0.33

Lastly, the BPD outcomes between the groups were not statistically different when using chi-square tests. Both the overall rates of BPD and the severity of BPD were similar between the two groups (Table 3).

**Table 3:** BPD outcomes ‡ n (%) BPD: bronchopulmonary dysplasia

	Intubated group (n=15)	Noninvasive group (n=14)	P-value
BPD ‡	9 (60)	7 (50)	0.59
BPD severity ‡			0.61
Grade 1	4 (27%)	4 (29%)	
Grade 2	5 (33%)	3 (21%)	
Grade 3	0 (0%)	0 (0%)	

## Discussion

The Edi catheter is used to monitor Edi during respiration. The catheter assesses neural respiratory control by measuring the total action potentials of the motor units in the diaphragmatic crura [13], which are representative of phrenic nerve activity and global diaphragm activation [14]. The Edi is depicted as a waveform with a cyclic pattern that has clear peaks and minimum values. The Edi minimum is the end-expiratory Edi value, while the Edi peak reflects the amplitude of electrical activity associated with the patient's inspiratory effort [15]. There are reference ranges for target Edi minimums and peaks in neonates while on NAVA mode; however, as a pure monitoring tool, there are no established reference ranges. The goal of this study was to evaluate Edi metrics as a monitoring tool for Edi while on other modes of ventilation. This is important because, at present, NAVA mode is not widely used in NICUs. In practice, we continue to choose modes of respiratory support based on subjective data, which leaves considerable room for variability in practice and outcomes. The results of our study indicate baseline differences between the groups. The group that was intubated had lower gestational age, birth weight, and Apgar scores at one minute after birth. This correlates with a likely sicker population with a higher risk of respiratory failure and long-term comorbidities.

**Figure 1:**
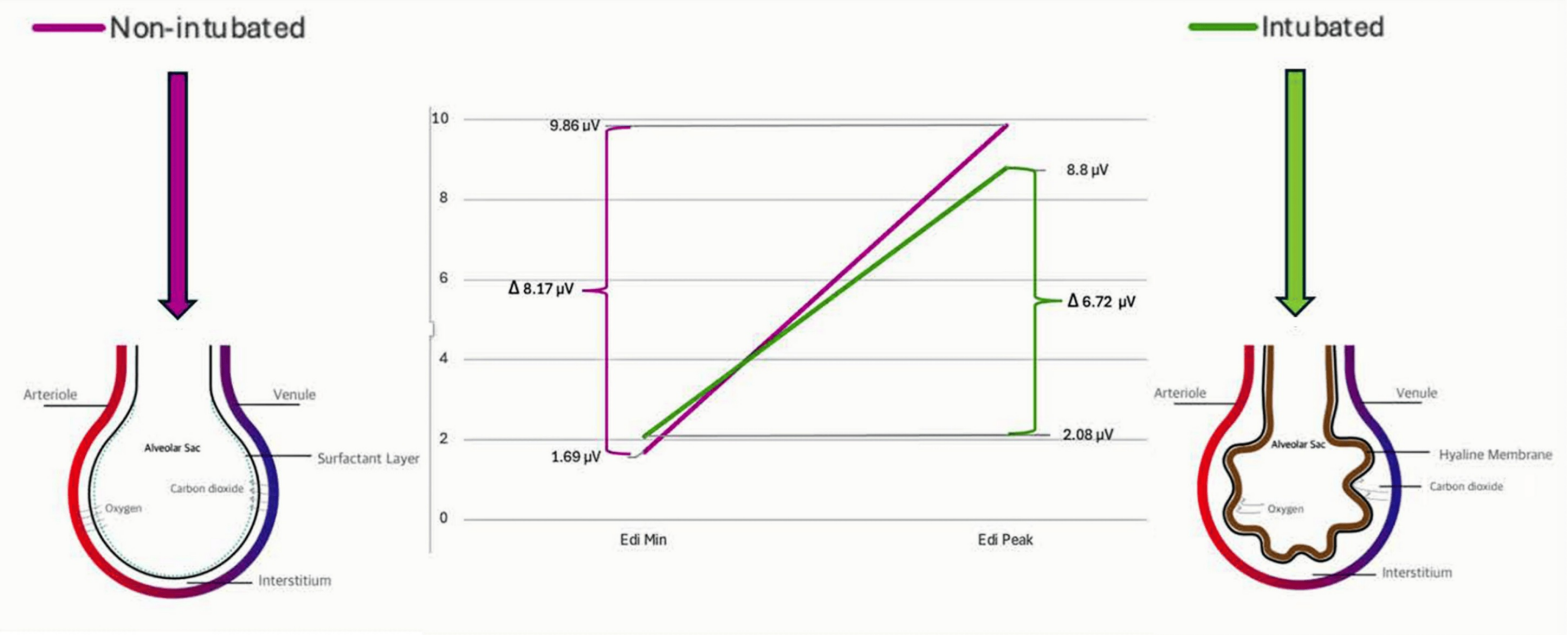
Correlation of Edi metrics with the degree of respiratory disease Edi: electrical activity of the diaphragm; min: minimum The figure is created by the authors.

As previously mentioned, it has been shown that worse respiratory illness is generally associated with sustained higher Edi metrics [11]. Our findings suggest that the infants who were intubated - presumably with worse respiratory disease - had higher Edi minimums, but lower delta Edi values and a trend towards lower Edi peaks. The Edi minimum correlates with the end-expiratory Edi value, or the diaphragmatic activity at the end of expiration. This can be thought of as the internal positive end-expiratory pressure (PEEP). Perhaps the higher Edi minimum in the sicker group may suggest the need for more signaling at the end of expiration to maintain adequate distending pressure to keep alveoli open in the setting of worse respiratory disease. In practice, smaller infants are generally placed on a PEEP of 5 cmH_2_O. It is possible that a higher Edi minimum indicates a potential need for more support at end-expiration (Figure 1). Determining the optimal PEEP remains a significant question, as there is insufficient evidence to guide PEEP level selection for preterm infants. More studies are needed to further evaluate how to determine the ideal PEEP for this population [16].

Given that the intubated group received more support, it is reasonable that the delta Edi was lower in this group. Delta Edi should theoretically correlate with the tidal volume generated with each breath. In practice, we adjust ventilator settings, such as peak inspiratory pressures or tidal volumes, based on gas measurements, increasing support with worsening respiratory acidosis. In the intubated group, the ventilator was set to support breaths and tidal volumes according to needs assessed by clinical evaluation of comfort and blood gases. Therefore, these subjects were responsible for less work in maintaining adequate tidal volume and ventilation, resulting in lower delta Edi and Edi peaks. Another possible explanation is that the higher tidal volume in the non-intubated group suggests less severe lung disease and a greater intrinsic ability to generate adequate tidal volume for ventilation, which could potentially be explained by greater gestational age and birth weight.

The overall goal in optimizing respiratory management is to minimize adverse outcomes, such as BPD. When comparing BPD outcomes between the groups, there were no differences in the overall rates of BPD or the severity of BPD. Given that the intubated group was presumably sicker and had lower gestational age and birth weight, it might be speculated that BPD rates and severity would be higher in this group. The absence of differences suggests that it is not only initial demographics and presentation that predict BPD outcomes, but also the overall clinical course over the subsequent days and weeks after birth that serves as a predictor.

This study has several limitations. First, because preterm infants requiring intubation are often intubated shortly after birth, it was difficult to quantify baseline Edi metrics to compare the two groups before any invasive intervention. Knowing baseline metrics would provide more information regarding disease severity. Furthermore, potential variability in Edi catheter placement and signal interpretation could introduce measurement bias. Additionally, this was a small, single-center pilot study. Therefore, while it provided some information regarding Edi metrics as a monitoring tool, it was not powered to predict target Edi metrics for this population, and its generalizability is limited. As this was an observational study, there was a lack of standardized ventilator settings or uniform clinical protocols across subjects, as that is not our standard practice. This likely affected the comparability of the groups. Nevertheless, the results indicate that there is utility in future studies to try to determine Edi metrics that would suggest adequate support, under-support, or over-support to help guide respiratory management.

## Conclusions

Optimal respiratory management remains a significant area of discussion in the field of neonatology. There is wide variation in practice regarding modes of ventilation, both invasive and noninvasive, with no clear best practice. Over-support runs the risk of unnecessary volutrauma and barotrauma, among other complications, while under-support risks atelectasis and subsequent inflammatory responses. We lack objective data and criteria to guide our practice. This study is the first to examine Edi metrics purely as a monitoring tool to assess the degree of disease severity and the sufficiency of support provided. The Edi catheter is a safe monitoring tool, and the results of this study suggest that its use may provide valuable data to help standardize and guide clinical practice. Larger, multicenter prospective studies would be helpful in further understanding the utility of Edi metrics to guide clinical management.
